# Neuroendoscopic Resection of Intraventricular Tumors and Cysts through a Working Channel with a Variable Aspiration Tissue Resector: A Feasibility and Safety Study

**DOI:** 10.1155/2013/471805

**Published:** 2013-06-13

**Authors:** Edjah Kweku-Ebura Nduom, Eric A. Sribnick, D. Ryan Ormond, Costas G. Hadjipanayis

**Affiliations:** ^1^Department of Neurosurgery, Emory University School of Medicine, 1365B Clifton Road NE, Suite 6200, Atlanta, GA 30322, USA; ^2^Winship Cancer Institute of Emory University, Atlanta, GA, USA

## Abstract

Pure neuroendoscopic resection of intraventricular lesions through a burr hole is limited by the instrumentation that can be used with a working channel endoscope. We describe a safety and feasibility study of a variable aspiration tissue resector, for the resection of a variety of intraventricular lesions. Our initial experience using the variable aspiration tissue resector involved 16 patients with a variety of intraventricular tumors or cysts. Nine patients (56%) presented with obstructive hydrocephalus. Patient ages ranged from 20 to 88 years (mean 44.2). All patients were operated on through a frontal burr hole, using a working channel endoscope. A total of 4 tumors were resected in a gross total fashion and the remaining intraventricular lesions were subtotally resected. Fifteen of 16 patients had relief of their preoperative symptoms. The 9 patients who presented with obstructive hydrocephalus had restoration of cerebrospinal fluid flow though one required a ventriculoperitoneal shunt. Three patients required repeat endoscopic resections. Use of a variable aspiration tissue resector provides the ability to resect a variety of intraventricular lesions in a safe, controlled manner through a working channel endoscope. Larger intraventricular tumors continue to pose a challenge for complete removal of intraventricular lesions.

## 1. Introduction

The resection of lesions within the ventricular system presents a challenge to neurosurgeons. The deep location of intraventricular tumors or cysts can lead to neurological sequelae before or after surgery. With the use of the operative microscope, most lesions of the lateral and third ventricles are accessed by a craniotomy and either a transcortical or interhemispheric transcallosal approach. These approaches are associated with brain retraction that can result in seizures, focal neurologic deficits, and cognitive impairment. A pure neuroendoscopic approach for the management of intraventricular lesions has been described and remains a minimally invasive approach that avoids brain retraction and provides direct lesion visualization [1, 2]. Access to the ventricular system through a burr hole and use of a working channel endoscope can permit endoscopic visualization of tumors and cysts that may be sampled or resected. Reestablishment of CSF communication pathways is also possible endoscopically when patients develop obstructive hydrocephalus due to their intraventricular pathology. The neuroendoscopic resection of intraventricular cysts and tumors is limited by the appropriate instrumentation that can be used with a working channel endoscope. Conventional instruments used through the working channel endoscope include cautery, grasping forceps, and aspiration devices. This has limited tumor and cyst resection through a working channel endoscope to piecemeal removal of intraventricular lesions [1]. Here we describe our initial experience with a 1.9 and 1.1 mm variable aspiration tissue resector used through the working channel endoscope for the resection of a variety of intraventricular tumors and cysts. Feasibility and safety were assessed in this series of patients.

## 2. Materials and Methods

Our initial experience using the variable aspiration tissue resector (NICO Myriad, NICO, Corp., Indianapolis, IN, USA) involves 16 patients (Table 1) with a variety of intraventricular pathologic lesions in the lateral (*n* = 8) or third ventricles (*n* = 8). Tumors or cysts treated include a pineal cyst, lateral ventricle arachnoid cysts (*n* = 3), a large colloid cyst, a benign mixed astroglial cyst, low-grade gliomas (*n* = 4) (1 myxopapillary ependymoma, 1 WHO grade II astrocytoma, 1 pilocytic astrocytoma, and 1 subependymal giant cell astrocytoma (SEGA)), a dysembryoplastic neuroepithelial-like tumor (DNET), an epidermoid tumor, an immature teratoma, a craniopharyngioma, a giant pituitary macroadenoma with intraventricular extension, and a pineal parenchymal tumor (intermediate differentiation). Patient ages ranged from 20 to 88 (mean 44.2). Nine patients (56%) presented with ventriculomegaly and obstructive hydrocephalus due to their intraventricular lesion. Five patients presented with memory difficulties, and two presented with seizures as part of their initial presentation. Fourteen out of 16 patients presented with progressive headaches.

All patients underwent neuroendoscopic resection through a single frontal burr hole. Neuronavigation (Medtronic StealthStation TREON) was used in all cases and registration of the working channel endoscope was performed. The majority (*n* = 14) of patients underwent neuroendoscopic resection utilizing a 30-degree Aesculap MINOP working channel endoscope (Aesculap Co., Tuttlingen, Germany). Two patients underwent surgery with use of the smaller diameter 30-degree Oi Handypro working channel endoscope (Karl Storz Co., Tuttlingen, Germany). A 1.9 mm diameter variable aspiration tissue resector was used with the Aesculap MINOP working channel endoscope and a 1.1 mm device was used with the Oi Handypro endoscope. All surgeries were performed by the senior surgeon (CH). Patient information for this study was collected with approval from the Institutional Review Board at Emory University. Extent of resection was calculated using the Osirix Open Source Imaging Software.

### 2.1. Technique

Each patient in the supine position was placed in a 3-pin Mayfield fixation device allowing for neutral positioning of the head. Neuronavigation was used for each patient to aid in cannulating the ventricular system and also for determining the proper trajectory to the intraventricular lesion. Registration of the tip of the working channel endoscope was also performed in all cases for navigation in the ventricular system and for the tumor or cyst resections. The right lateral ventricle was cannulated in 13 cases, and the left lateral ventricle in 3 cases. 

A 2 cm frontal vertical incision was made 3 cm lateral to midline in the region of the coronal suture. A single burr hole was placed with a high-speed drill measuring at least 7 mm. After opening the dura and cauterization, a 19 or 12 French peel-away sheath catheter was passed into the lateral ventricle at 5-6 cm with neuronavigation and secured in position. The Xomed Endo Scrub (Medtronic Inc.) irrigation system was attached to a port on the working channel of the endoscope in addition to suction. A high-definition (1080 p) camera head and light source were used for illumination and visualization.

**Figure 1:**
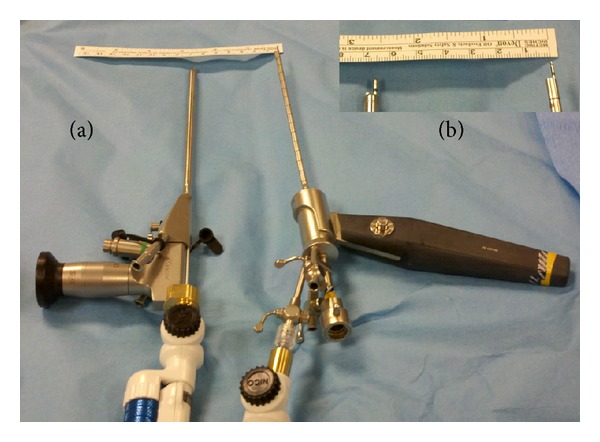
The NICO Myriad variable aspiration tissue resector. (a) On the left, the 1.9 mm device has been placed through the working channel of the Aesculap MINOP endoscopic system. On the right, the 1.1 mm device has been placed through the working channel of the Storz IO endoscopic system. (b) A closeup of the resecting tips extending beyond the endoscopes, showing the size of the complete system within the ventricle.

After insertion of the neuroendoscope working channel and identification of the intraventricular lesion, cautery of the tumor or cyst capsule was performed through the working channel. The variable aspiration tissue resector was subsequently placed through the working channel of the endoscope and secured in place with a tightening screw (Figure 1). The depth of insertion and rotation of the aperture of the resector was controlled with use of thumb dials on the device. Tissue resection was performed with the foot pedal control, and the intensity of aspiration and resection could be set with the console.

Any bleeding encountered during each neuroendoscopic procedure was controlled with irrigation or bipolar cautery through the working channel of the endoscope. 

## 3. Results

### 3.1. Hospitalization, Follow-Up, and Symptom Resolution

The median length of hospitalization was 7 days. Eleven patients were discharged home, three to acute rehabilitation, and two patients to assisted living facilities. One patient died 135 days after surgery from complications related to diabetes insipidus. The median clinical followup was 4.35 months.

Fifteen patients (94%) had relief of their preoperative symptoms. Fourteen patients (88%) had preoperative headaches that improved after surgery. Eight of nine patients who presented with ventriculomegaly and obstructive hydrocephalus had ventricular decompression and restoration of cerebrospinal fluid flow without ventriculoperitoneal shunt (VPS) placement. One patient without hydrocephalus presented with simple partial seizures, which resolved after surgery. 

### 3.2. Extent of Cyst or Tumor Resection

All three arachnoid cysts and the pineal cyst were partially resected. Of the intraventricular tumors, the large colloid cyst, epidermoid tumor including its capsule, immature teratoma, and the benign mixed astroglial cyst were resected in a gross total fashion. The remaining 8 intraventricular tumors were partially resected (Table 2). The pineal parenchymal tumor patient underwent fractionated intensity modulated radiotherapy (IMRT) for treatment of her residual tumor leading to complete resolution of her lesion ten months after treatment. 

Three patients were taken back to the operating room for a repeat neuroendoscopic approach to further resect their residual intraventricular tumor (one with a SEGA; one with a DNET; and one with a teratoma) and reestablish cerebral spinal fluid flow communication to avoid placement of a VPS. In all three patients, during the initial procedure, neuroendoscopic resection with the variable aspiration tissue resector was stopped prematurely due to visualization problems after tumor bleeding. The intraventricular hemorrhage noted intraoperatively did not require conversion to an open craniotomy for hematoma evacuation in any of the patients. All three patients remained neurologically stable after their initial neuroendoscopic tumor resection. Placement of an EVD permitted clearing of blood from the ventricles prior to their second procedure. After discharge, no tumor or cyst has demonstrated recurrence or further needs for any surgical management.

### 3.3. Restoration of CSF Communication Pathways

All patients with intraventricular cysts had restoration of CSF communication pathways with resolution of their obstructive hydrocephalus. No patients with intraventricular cysts required placement of a VPS. Restoration of CSF communication pathways was achieved in all tumor patients except for patient 2 who required placement of a VPS for persistent hydrocephalus and treatment of a pseudomeningocele. One patient was taken back to surgery for an endoscopic third ventriculostomy (ETV) and lysis of ventricular adhesions after inability to wean her EVD. Ten patients had an ETV performed at the time of their initial surgery, and two additional patients had an ETV performed in a subsequent case. Fourteen out of 16 patients had septum pellucidum fenestrations.

## 4. Illustrative Cases

### 4.1. Patient 13 (Arachnoid Cyst) (See Video 1 in the Supplementary Material Available Online at http://dx.doi.org/10.1155/2013/471805)

**Figure 2:**
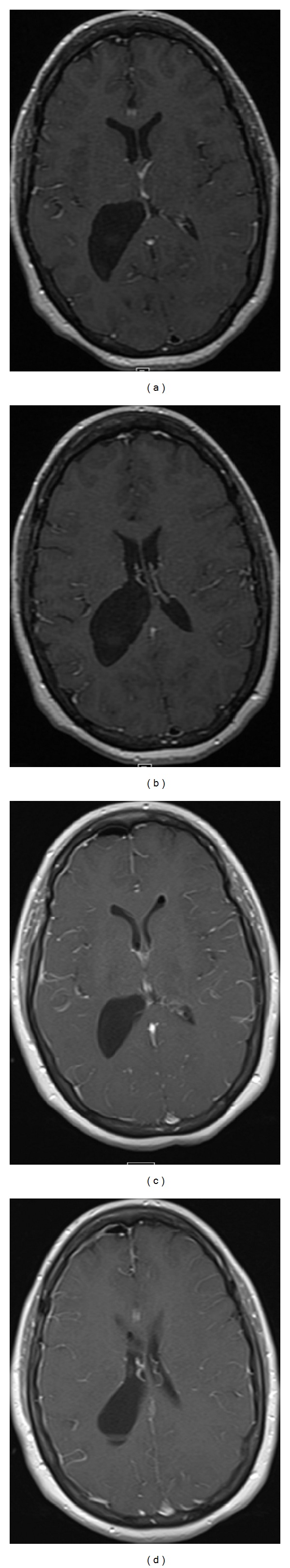
Patient 1, lateral ventricle arachnoid cyst. Preoperative ((a) and (c)) and postoperative ((b) and (d)) contrast enhanced axial T1-weighted magnetic resonance imaging, demonstrating decompression of the cyst and lateral ventricles.

A 35-year-old patient with no previous history of headaches presented with one month of progressive severe headaches. A CT scan, followed by an MRI of the brain, demonstrated a right lateral ventricle arachnoid cyst and associated ventriculomegaly of the right lateral ventricle. After three months of conservative medical management, the patient's headaches were persistent and associated with dizzy spells. A repeat MRI demonstrated unchanged findings of the right lateral ventricle arachnoid cyst and associated ventriculomegaly. The patient elected to proceed with a neuroendoscopic exploration and potential resection of her arachnoid cyst. With the variable aspiration tissue resector, the arachnoid cyst capsule was drawn into the side cutting aperture from the ependymal surface and partially resected, permitting reestablishment of CSF flow. The patient was discharged on postoperative day four without incident. Postoperative MRI demonstrated a reduction in ventricular size, and this remained stable at three-month follow-up (Figure 2). The patient had resolution of her severe presenting headaches.

### 4.2. Patient 14 (Pilocytic Astrocytoma) (Video 2)

**Figure 3:**
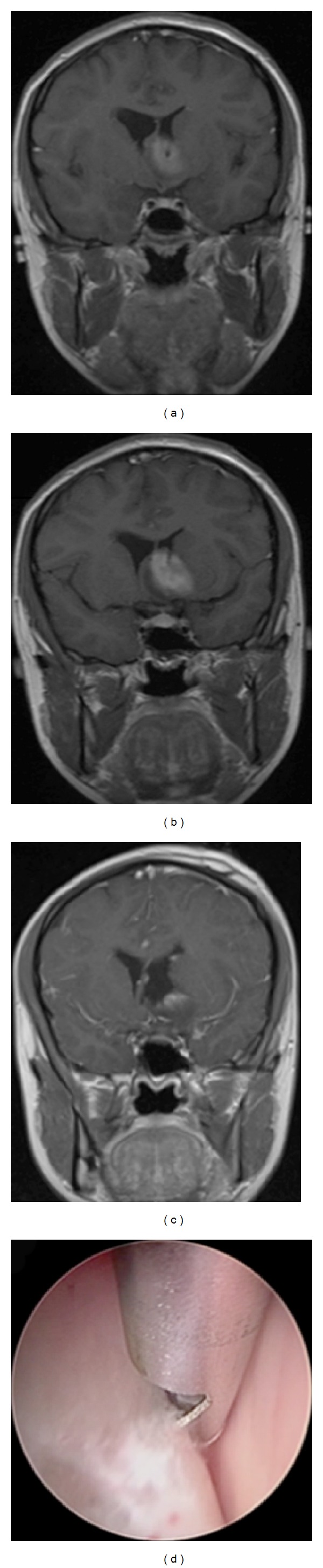
Patient 14, pilocytic astrocytoma. ((a) and (b)) Preoperative coronal T1-weighted contrast-enhanced magnetic resonance imaging showing enhancing lesion and obstructive hydrocephalus. (c) Decrease in ventricular size with interval debulking of lesion. (d) Intraoperative endoscopic view of the tissue aspirator resecting tumor.

Patient 14 is a 20-year-old female who woke up the day of presentation with a severe headache. Both CT and MR images (Figure 3) were obtained revealing a left lateral ventricular lesion extending from the hypothalamic region. The patient was initially alert but became lethargic requiring EVD placement. The patient underwent a subtotal resection of her pilocytic astrocytoma with the variable aspiration tissue resector through the working channel endoscope (Figure 3). The patient's EVD was weaned successfully on post-operative day four, and she was discharged home on post-operative day seven, neurologically intact. 

### 4.3. Patient 15 (Large Colloid Cyst) (Video 3)

**Figure 4:**
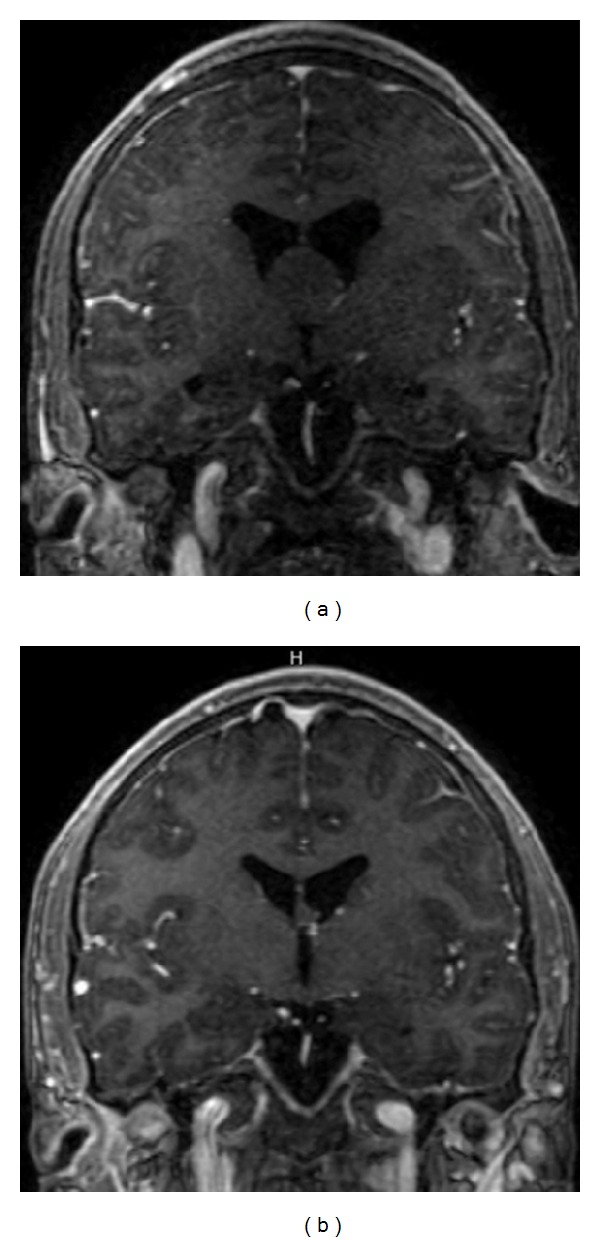
Patient 15, large colloid cyst. (a) Preoperative contrast enhanced coronal T1-weighted magnetic resonance imaging (MRI) showing a lesion with obstructive hydrocephalus. (b) 3-month follow-up MRI shows gross total resection of lesion and resolution of obstructive hydrocephalus.

Patient 15 was a 20-year-old male presenting with progressive headache two days following an episode of transient confusion and word-finding difficulty. CT scan of the head demonstrated a 2.3 cm third ventricular cystic lesion. An MRI confirmed the suspected diagnosis of a colloid cyst (Figure 4), and a neuroendoscopic resection of the mass was performed. Initially, endoscopic cautery of the colloid cyst capsule was performed to shrink the colloid cyst permitting dissection off the roof of the third ventricle and the fornix. Due to the large size of the colloid cyst, en block resection was not possible. Evacuation of the contents of the colloid cyst was first performed followed by complete resection of cyst capsule with the variable aspiration tissue resector (Figure 4).

## 5. Discussion

### 5.1. Microsurgical Approaches to Intraventricular Lesions

Use of a craniotomy and a transcallosal or transcortical microsurgical approach provides access to intraventricular pathology for resection purposes. These commonly used approaches have the advantage of allowing the surgeon to perform bimanual dissection with the microscope for tumor or cyst resection using a wide range of microscopic instruments and bipolar cautery. Microsurgical approaches to intraventricular lesions after a craniotomy can be associated with significant neurologic deficits due to brain retraction and possibly increased seizure risk postoperatively [3–6]. Others have described the use of tubular retractors in pediatric and adult patient populations for deep-seated lesions, but with limited experience with intraventricular lesions [7, 8]. 

### 5.2. Endoscopic Approaches to Intraventricular Lesions

There have been multiple reports of the resection of intraventricular lesions using a pure endoscopic approach with conventional working channel instruments, including suction, grasping forceps, and cutting instruments [1]. Souweidane and Luther reported the resection of 7 solid intraventricular brain tumors and outlined the difficulties associated with resecting these lesions given the restrictive instruments available to them at that time [2]. Their experience was also similar to that of Gaab and Schroeder who reported the purely endoscopic resection of intraventricular lesions [9]. In both series, the attempted resection of solid lesions with diameters greater than 20 mm was extremely difficult due to the small working channels of the endoscopes used and the length of surgery required in these cases.

The endoscope has also been used for assistance and visualization of deep structures while using a bimanual conventional open surgical technique. Interhemispheric endoscopic-assisted approaches have been reported, but this requires a large craniotomy and access near the superior sagittal sinus [10]. Mclaughlin et al. recently evaluated the use of a port-assisted endoscopic technique for the resection of intraventricular lesions, allowing the use of bimanual technique [11]. This approach requires a craniotomy and placement of a 1.2 cm port through the brain to the tumor or cyst and use of a nonworking channel endoscope for visualization.

### 5.3. Previously Reported Use of Variable Aspiration Tissue Resectors

There have been limited reported case series on the use of variable-aspiration tissue resectors for the resection of intraventricular lesions. Lekovic et al. documented the use of a previous version to the current device in the resection of two hypothalamic hamartomas through a working channel endoscope [12]. Several studies have been performed on the use of the current variable aspiration tissue resector. Mohanty et al. described the sub- or near-total resection of large intraventricular tumors (two craniopharyngiomas and one subependymoma) [13]. Albright and Okechi described the resection of two pineal lesions without followup [14]. The two largest series to date were reported by Sood et al. and Dlouhy et al. [15, 16]. Sood et al. described their use of the device in resecting 23 lesions including brain and spinal lesions with good short-term follow-up results [15]. Dlouhy et al. describe their experience with the variable-aspiration tissue resector in fifteen patients [16]. These series, as with our series, all describe the benefits and limitations of the device, but our series is the largest to quantify extent of resection and how this relates to the use of the variable-aspiration tissue resector. 

### 5.4. Strengths, Limitations, and Safety

The ability to rotate the aperture and lengthen or shorten the length of the variable aspiration tissue resector permitted safe resection of all lesions described in this series. Proper visualization of the aperture and placement away from neurovascular structures permitted controlled tissue resection with the foot pedal control. The console could be adjusted for greater or lesser aspiration and resection. In our limited experience, the variable aspiration tissue resector seemed to work best on soft tumors with minimal vascularity. One of the four tumors completely resected was a large colloid cyst, but, in our experience, colloid cysts can typically be resected without the use of the variable aspiration tissue resector. With larger cysts (>2 cm), rapid debulking of the cyst contents and complete resection of the capsule can be performed well with the variable aspiration tissue resector.

More vascular tumors, such as gliomas, were amenable to subtotal resection in our initial experience, which was often the goal of surgery. However, cautery is not provided by the variable aspiration tissue resector. Tumor resection was halted intermittently for hemostasis with irrigation and endoscopic cautery through the working channel. Use of multiple channels simultaneously has been reported with the working channel endoscope to optimize lesion resection [17]. We felt that the introduction of endoscopic cautery through a separate working channel with the variable aspiration tissue resector in place resulted in visual obstruction during tumor resection. 

Due to inadequate hemostasis with the endoscopic cautery and poor endoscopic visualization from blood products in the ventricular system, three patients required repeat endoscopic operations for further tumor resection. We have found that adequate tumor capsule cautery prior to neuroendoscopic resection with the variable aspiration tissue resector may reduce bleeding from the residual tumor that may halt the surgery prematurely. While we did not have to convert to a craniotomy for evacuation of an intraventricular hematoma, aggressive resection with the variable aspiration tissue resector can result in intraoperative bleeding which may require an emergent craniotomy for definitive control. The development of newer bipolar cautery instruments that can be used through the working channel endoscope may provide the ability to better cauterize tumor capsules and intratumoral bleeding during resection with the variable aspiration tissue resector. 

While we were able to completely resect one immature teratoma with a diameter of 29 mm, the remainder of lesions greater than 20 mm were subtotally resected. We did achieve our goal of significant debulking of these lesions with restoration of CSF flow in all but one of the cases, even when dealing with lesions with diameters up to 36 mm. A craniotomy and microsurgical technique may have precluded the need for neuroendoscopic reoperation in three cases, but the stated preoperative goal of subtotal resection was obtained in all cases without the need for conversion to an open craniotomy.

## 6. Conclusions

In summary, the variable aspiration tissue resector can be safely utilized for the resection of a variety of solid tumors or cysts involving the ventricular system through a working channel endoscope. This approach remains limited by difficulties in controlling bleeding encountered while resecting more vascularized lesions, and in maneuverability to visualize lesions greater than 2 cm.

## Supplementary Material

Supplementary Materials: Intraoperative videos of illustrative cases are included here.
Video 1: Demonstrates the resection of an arachnoid cyst, Patient 13.
Video 2: Demonstrates the resection of the pilocytic astrocytoma, Patient 14.
Video 3: Demonstrates the evacuation of the contents of a large colloid cyst, Patient 15.

## Figures and Tables

**Table 1:** Patient characteristics.

Patient number	Age	Sex	Disposition	Obstructive HCP	Diagnosis	Outcome
Patient 1	37	M	Home	No	Lateral ventricular arachnoid cyst	Improvement of headaches, discharged home
Patient 2	30	M	Rehab	Yes	3rd ventricular immature teratoma	Underwent suboccipital craniotomy for pineal region immature teratoma. VPS placed for communicating hydrocephalus. Patient died from complications resulting from diabetes insipidus (DI)
Patient 3	71	F	SNF	No	Third ventricular epidermoid	Headaches improved, discharged for rehabilitation
Patient 4	43	F	Home	No	Lateral ventricular arachnoid cyst	Resolution of headaches, discharged home
Patient 5	27	F	Home	Yes	Pineal region cyst	Resolution of headaches, discharged home
Patient 6	72	F	Home	No	Lateral ventricular mixed astroglial cyst	Improvement of headaches, discharged home
Patient 7	88	F	SNF	Yes	Ventricular astrocytoma	Resolution of gait difficulties, discharged for rehabilitation, now resides in assisted living facility
Patient 8	47	M	Rehab	Yes	Ventricular/suprasellar pituitary adenoma	Had endoscopic transsphenoidal approach for further resection of giant pituitary adenoma, discharged for rehabilitation, headaches improved
Patient 9	47	M	Home	Yes	Hypothalamic myxopapillary ependymoma	Resolution of headaches, discharged home
Patient 10	40	F	Rehab	Yes	Pineal parenchymal tumor of intermediate differentiation	Headaches, gait difficulties, and diplopia resolved, discharged for rehabilitation. Received postoperative IMRT
Patient 11	20	M	Home	No	Subependymal giant-cell astrocytoma	Patient required repeat resection and ETV, had resolution of headaches, nausea and vomiting, and then discharged home
Patient 12	32	M	Home	No	Dysembryoplastic neuroepithelial-like tumor	Resolution of seizures, discharged home, now off of antiepileptic medications
Patient 13	36	F	Home	Yes	Lateral ventricular arachnoid cyst	Headaches improved, discharged home
Patient 14	20	F	Home	Yes	Pilocytic astrocytoma	Headaches improved, discharged home
Patient 15	20	M	Home	Yes	Colloid Cyst	Resolution of headaches, discharged home
Patient 16	77	M	Home	No	Craniopharyngioma	Headaches improved, discharged home

ETV: endoscopic third ventriculostomy; VPS: ventriculoperitoneal shunt.

**Table 2:** Extent of resection.

Patient no.	Diagnosis	Procedure	Extent of resection
Patient 1	Lateral ventricular arachnoid cyst	Cyst fenestration, ETV, SPF	Fenestration
Patient 2	3rd ventricular immature teratoma	Resection, ETV, SPF	Gross total resection
Patient 3	Third ventricular epidermoid	Resection, ETV, SPF	Gross total resection
Patient 4	Lateral ventricular arachnoid cyst	Cyst fenestration, ETV, SPF	Fenestration
Patient 5	Pineal region cyst	Resection, ETV, SPF	Gross total resection
Patient 6	Lateral ventricular mixed astroglial cyst	Resection, ETV, SPF	Gross total resection
Patient 7	Ventricular astrocytoma	Subtotal resection, ETV, SPF	22% resection
Patient 8	Ventricular/suprasellar pituitary adenoma	Subtotal resection, SPF	Postop MRI not available
Patient 9	Hypothalamic myxopapillary ependymoma	Resection, ETV, SPF	75% resection
Patient 10	Pineal parenchymal tumor of intermediate differentiation	Biopsy, ETV	22% resection
Patient 11	Subependymal giant-cell astrocytoma	Resection, SPF	92% resection
Patient 12	Dysembryoplastic neuroepithelial-like tumor	Subtotal resection, ETV, SPF	36% resection
Patient 13	Lateral ventricular arachnoid cyst	Cyst fenestration, SPF	Fenestration
Patient 14	Pilocytic astrocytoma	Subtotal resection, SPF	61% resection
Patient 15	2 cm colloid cyst	Subtotal resection, SPF	Gross total resection
Patient 16	Craniopharyngioma	Subtotal resection, ETV, SPF	53.6% resection

ETV: endoscopic third ventriculostomy; SPF: septum pellucidum fenestration.
